# 1-Benzoyl-3-(4-hy­droxy­phen­yl)thio­urea

**DOI:** 10.1107/S1600536810045988

**Published:** 2010-11-13

**Authors:** Aisha A. Al-abbasi, Siew San Tan, Mohammad B. Kassim

**Affiliations:** a School of Chemical Sciences and Food Technology, Faculty of Science and Technology, Universiti Kebangsaan Malaysia, UKM 43600 Bangi Selangor, Malaysia

## Abstract

In the title compound, C_14_H_12_N_2_O_2_S, the amino­phenol and the benzoyl groups adopt a *syn–anti* configuration with respect to the thiono C=S group across the thio­urea C—N. The dihedral angle between the mean planes of the benzoyl and hy­droxy­phenyl rings is 36.77 (8)°. The mol­ecules are stabilized by intra­molecular N—H⋯O hydrogen bonds. In the crystal, weak inter­molecular C—H⋯O, O—H⋯S and N—H⋯O hydrogen bonds link the mol­ecules into a chain along the *c* axis.

## Related literature

For the preparation and chemical properties of related compounds, see: Zhang *et al.* (2001 ▶). For related structures, see: Abosadiya *et al.* (2007 ▶); Hung *et al.* (2010 ▶); Yamin & Yusof (2003 ▶). For bond-length data, see: Allen *et al.* (1987 ▶).
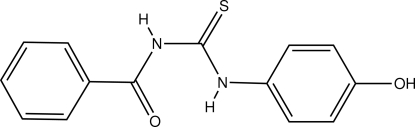


## Experimental

### 

#### Crystal data

C_14_H_12_N_2_O_2_S
*M* *_r_* = 272.33
Orthorhombic, 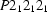

*a* = 5.5865 (10) Å
*b* = 14.451 (2) Å
*c* = 16.462 (3) Å
*V* = 1329.0 (4) Å^3^
*Z* = 4
Mo *K*α radiation
μ = 0.24 mm^−1^
*T* = 298 K
0.50 × 0.48 × 0.35 mm

#### Data collection

Bruker SMART APEX CCD area-detector diffractometer
Absorption correction: multi-scan (*SADABS*; Bruker, 2000 ▶) *T*_min_ = 0.886, *T*_max_ = 0.919
7608 measured reflections
2351 independent reflections
2143 reflections with *I* > 2σ(*I*)
*R*_int_ = 0.018

#### Refinement

*R*[*F* ^2^ > 2σ(*F* ^2^)] = 0.031
*wR*(*F* ^2^) = 0.085
*S* = 1.05
2351 reflections
173 parameters
H-atom parameters constrained
Δρ_max_ = 0.13 e Å^−3^
Δρ_min_ = −0.14 e Å^−3^
Absolute structure: Flack (1983 ▶), 958 Friedel pairs
Flack parameter: 0.09 (9)

### 

Data collection: *SMART* (Bruker, 2000 ▶); cell refinement: *SAINT* (Bruker, 2000 ▶); data reduction: *SAINT*; program(s) used to solve structure: *SHELXS97* (Sheldrick, 2008 ▶); program(s) used to refine structure: *SHELXL97* (Sheldrick, 2008 ▶); molecular graphics: *SHELXTL* (Sheldrick, 2008 ▶); software used to prepare material for publication: *SHELXTL*, *PARST* (Nardelli, 1995 ▶) and *PLATON* (Spek, 2009 ▶).

## Supplementary Material

Crystal structure: contains datablocks I, global. DOI: 10.1107/S1600536810045988/jj2068sup1.cif

Structure factors: contains datablocks I. DOI: 10.1107/S1600536810045988/jj2068Isup2.hkl

Additional supplementary materials:  crystallographic information; 3D view; checkCIF report

## Figures and Tables

**Table 1:** Hydrogen-bond geometry (Å, °)

*D*—H⋯*A*	*D*—H	H⋯*A*	*D*⋯*A*	*D*—H⋯*A*
N2—H2*B*⋯O1	0.86	1.95	2.631 (2)	135
N1—H1*B*⋯O2^i^	0.86	2.29	3.109 (2)	158
O2—H2*C*⋯S1^ii^	0.82	2.53	3.1533 (18)	134
C1—H1*A*⋯O2^i^	0.93	2.51	3.429 (3)	172
C11—H11*A*⋯O1^iii^	0.93	2.43	3.262 (2)	149

Symmetry codes: (i) 
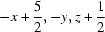
; (ii) 
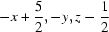
; (iii) 
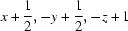
.

**Fig. 1.**
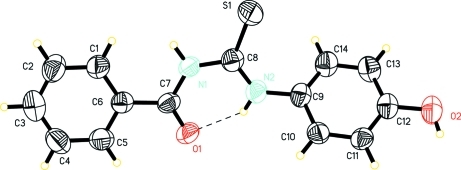
The molecular structure of (I), with displacement ellipsods drawn at the 50% probability level. Dashed lines indicate intramolecular N—H···O hydrogen bonds.

**Fig. 2.**
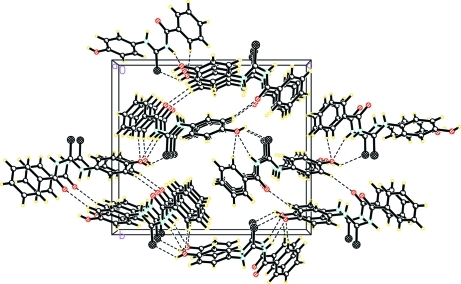
Crystal packing of (I) viewed down the a axis. Dashed lines indicate weak C—H···O, C—H···O, O—H···S and N—H···O hydrogen bonds.

## supplementary crystallographic information

### Comment

The title compound, I, is closely related to the previously reported *N*-benzoyl-*N'*-(3-hydroxyphenyl)thiourea (II) (Abosadiya *et al.*, 2007) compound. As in most benzoylthiourea derivatives of the type *R*_1_C(O)NHC(S)NH*R*_2_, the title compound has a *syn–anti* configuration for the hydroxyphenyl and benzoyl groups with respect to the thiono C=S bond across the thiourea C—N bond (Fig 1). The bond lengths and angles in the molecules are in normal ranges (Allen *et al.*, 1987) and comparable to those in (II). All C—N bond lengths are shorter than the normal value for C—N single bond, and the bond lengths C7—O1 and C8—S1 become longer than normal values for a double bond, which suggests the presence of delocalized π-electrons. For example, the C=S bond (1.671 (2) Å) and C—N bond lengths (C8—N1 = 1.388 (2) Å, C8—N2 = 1.328 (2) Å and C9—N2 = 1.428 (2) Å) in the title compound are longer than that observed in an unsubsituted phenyl ring (III - Yamin *et al.*, 2003) in which the C=S bond length (1.6567 (15) Å) and C—N bond lengths are 1.393 (2), 1.326 (2) and 1.408 (2) Å, respectively. This is due to donating effect of OH group in the *para* position, which contributes to an increase in the bond length.

The benzoyl ring [C1/C2/C3/C4/C5/C6/C7/O1] (A), hydroxyphenyl ring [N2/C9/C10/C11/C12/C13/C14/O2] (B) and thiourea [(S1/N1/N2/C8/] (C) fragments are essentially planar with maximum deviations from their mean planes of 0.045 (2) Å for atom O1 (A), 0.010 (2) Å for atom C10 (B) and 0.008 (2)Å for atom C8 (C), respectively. The dihedral angle between the mean planes of A and B is 36.77 (8)°. The crytal structure is stabilized by an intramolecular N2—H2B···O1 hydrogen bond which forms a six-membered ring (N2/H2B/O1/C7/N1/C8) commonly observed in this class of ligands. In addition, the molecules are linked by weak intermolecular C11—H···O1, C1—H···O2, O2—H···S1 and N1—H···O2 hydrogen bonds (Table 2), resulting in a one-dimensional chain along the *c*-axis (Fig 2).

### Experimental

The title compound was first synthesized by (Zhang *et al.*, 2001) under the condition of solid-liquid phase transfer catalysis using polyethylene glycol as catalyst, however, a much simpler method was used to synthesize the title compound. The reaction scheme involved a reaction of benzoyl chloride (10 mmol) with ammonium thiocyanate (10 mmol) in dry acetone. The product, benzoyl isothiocyanate was reacted with 4-hydroxy aniline (10 mmol) to give the title compound with a 56% yield. A slow evaporation of ethanolic solution of the compound gave light brawn crystals suitable for X-ray diffraction.

### Refinement

All the non hydrogen atom were refined anisotropically. the hydrogen positions were calculated to give an idealized geometry fixed to ride on their respective atoms, with *U*_iso_=1.2*U*_eq_ (C) for aromatic (CH = 0.93 Å), *U*_iso_=1.2*U*_eq_ (N) for N (NH = 0.86 Å) and *U*_iso_=1.5*U*_eq_ (O) for OH (OH = 0.82 Å).

### Crystal data

**Table:**

C_14_H_12_N_2_O_2_S	*F*(000) = 568
*M**_r_* = 272.33	*D*_x_ = 1.361 Mg m^−^^3^
Orthorhombic, *P*2_1_2_1_2_1_	Mo *K*α radiation, λ = 0.71073 Å
Hall symbol: P 2ac 2ab	Cell parameters from 1024 reflections
*a* = 5.5865 (10) Å	θ = 1.9–25.0°
*b* = 14.451 (2) Å	µ = 0.24 mm^−^^1^
*c* = 16.462 (3) Å	*T* = 298 K
*V* = 1329.0 (4) Å^3^	Block, brown
*Z* = 4	0.50 × 0.48 × 0.35 mm

### Data collection

**Table:**

Bruker SMART APEX CCD area-detector diffractometer	2351 independent reflections
Radiation source: fine-focus sealed tube	2143 reflections with *I* > 2σ(*I*)
graphite	*R*_int_ = 0.018
ω scans	θ_max_ = 25.0°, θ_min_ = 1.9°
Absorption correction: multi-scan (*SADABS*; Bruker, 2000)	*h* = −6→6
*T*_min_ = 0.886, *T*_max_ = 0.919	*k* = −15→17
7608 measured reflections	*l* = −19→17

### Refinement

**Table:**

Refinement on *F*^2^	Secondary atom site location: difference Fourier map
Least-squares matrix: full	Hydrogen site location: inferred from neighbouring sites
*R*[*F*^2^ > 2σ(*F*^2^)] = 0.031	H-atom parameters constrained
*wR*(*F*^2^) = 0.085	*w* = 1/[σ^2^(*F*_o_^2^) + (0.0494*P*)^2^ + 0.1346*P*] where *P* = (*F*_o_^2^ + 2*F*_c_^2^)/3
*S* = 1.05	(Δ/σ)_max_ = 0.001
2351 reflections	Δρ_max_ = 0.13 e Å^−^^3^
173 parameters	Δρ_min_ = −0.14 e Å^−^^3^
0 restraints	Absolute structure: Flack (1983), 958 Friedel pairs
Primary atom site location: structure-invariant direct methods	Flack parameter: 0.09 (9)

### Special details

**Table:**

Geometry. All e.s.d.'s (except the e.s.d. in the dihedral angle between two l.s. planes) are estimated using the full covariance matrix. The cell e.s.d.'s are taken into account individually in the estimation of e.s.d.'s in distances, angles and torsion angles; correlations between e.s.d.'s in cell parameters are only used when they are defined by crystal symmetry. An approximate (isotropic) treatment of cell e.s.d.'s is used for estimating e.s.d.'s involving l.s. planes.
Refinement. Refinement of *F*^2^ against ALL reflections. The weighted *R*-factor *wR* and goodness of fit *S* are based on *F*^2^, conventional *R*-factors *R* are based on *F*, with *F* set to zero for negative *F*^2^. The threshold expression of *F*^2^ > σ(*F*^2^) is used only for calculating *R*-factors(gt) *etc*. and is not relevant to the choice of reflections for refinement. *R*-factors based on *F*^2^ are statistically about twice as large as those based on *F*, and *R*- factors based on ALL data will be even larger.

### Fractional atomic coordinates and isotropic or equivalent isotropic displacement parameters (Å^2^)

**Table:**

	*x*	*y*	*z*	*U*_iso_*/*U*_eq_	
S1	1.13932 (14)	−0.04354 (4)	0.71251 (4)	0.0812 (2)	
O1	0.7785 (3)	0.23667 (10)	0.72476 (9)	0.0719 (4)	
N1	0.8692 (3)	0.09206 (10)	0.76731 (8)	0.0518 (4)	
H1B	0.8429	0.0530	0.8057	0.062*	
N2	1.0996 (3)	0.12902 (11)	0.65596 (9)	0.0565 (4)	
H2B	1.0469	0.1841	0.6646	0.068*	
C1	0.5090 (4)	0.10914 (14)	0.89160 (13)	0.0632 (5)	
H1A	0.6077	0.0573	0.8928	0.076*	
C2	0.3268 (5)	0.11715 (16)	0.94751 (14)	0.0712 (6)	
H2A	0.3032	0.0712	0.9862	0.085*	
C3	0.1812 (4)	0.19276 (18)	0.94584 (14)	0.0726 (6)	
H3A	0.0581	0.1983	0.9836	0.087*	
C4	0.2149 (4)	0.26068 (17)	0.88890 (14)	0.0734 (6)	
H4A	0.1140	0.3119	0.8879	0.088*	
C5	0.3978 (4)	0.25336 (14)	0.83317 (12)	0.0632 (5)	
H5A	0.4208	0.3000	0.7950	0.076*	
C6	0.5475 (3)	0.17701 (12)	0.83367 (10)	0.0487 (4)	
C7	0.7395 (4)	0.17240 (12)	0.77127 (10)	0.0512 (4)	
C8	1.0373 (4)	0.06508 (13)	0.70994 (11)	0.0527 (4)	
C9	1.2439 (4)	0.11670 (13)	0.58521 (11)	0.0504 (4)	
C10	1.1577 (4)	0.15191 (12)	0.51275 (11)	0.0520 (5)	
H10A	1.0118	0.1829	0.5119	0.062*	
C11	1.2858 (4)	0.14146 (12)	0.44198 (11)	0.0518 (5)	
H11A	1.2257	0.1643	0.3933	0.062*	
C12	1.5042 (3)	0.09692 (12)	0.44377 (12)	0.0515 (5)	
C13	1.5928 (4)	0.06247 (16)	0.51603 (11)	0.0596 (5)	
H13A	1.7399	0.0323	0.5170	0.072*	
C14	1.4626 (4)	0.07290 (16)	0.58682 (12)	0.0594 (5)	
H14A	1.5229	0.0503	0.6356	0.071*	
O2	1.6406 (3)	0.08472 (10)	0.37487 (9)	0.0682 (4)	
H2C	1.5717	0.1079	0.3359	0.102*	

### Atomic displacement parameters (Å^2^)

**Table:**

	*U*^11^	*U*^22^	*U*^33^	*U*^12^	*U*^13^	*U*^23^
S1	0.1110 (5)	0.0497 (3)	0.0828 (4)	0.0182 (3)	0.0277 (4)	0.0010 (3)
O1	0.1018 (11)	0.0536 (8)	0.0603 (8)	0.0184 (8)	0.0223 (8)	0.0179 (6)
N1	0.0712 (10)	0.0432 (8)	0.0410 (7)	0.0061 (8)	0.0053 (7)	0.0030 (6)
N2	0.0712 (10)	0.0480 (8)	0.0504 (8)	0.0014 (8)	0.0101 (8)	−0.0027 (7)
C1	0.0732 (14)	0.0513 (11)	0.0649 (12)	0.0051 (10)	0.0142 (10)	0.0055 (9)
C2	0.0783 (15)	0.0630 (13)	0.0723 (13)	−0.0108 (12)	0.0191 (12)	0.0028 (11)
C3	0.0575 (13)	0.0890 (17)	0.0713 (13)	−0.0058 (12)	0.0105 (11)	−0.0166 (13)
C4	0.0649 (14)	0.0824 (16)	0.0728 (14)	0.0219 (12)	0.0005 (12)	−0.0090 (12)
C5	0.0755 (14)	0.0596 (12)	0.0546 (11)	0.0119 (11)	−0.0026 (11)	0.0023 (9)
C6	0.0573 (11)	0.0472 (10)	0.0416 (8)	0.0009 (8)	−0.0045 (8)	−0.0040 (7)
C7	0.0669 (11)	0.0461 (10)	0.0406 (9)	0.0026 (9)	−0.0032 (8)	0.0018 (7)
C8	0.0627 (11)	0.0508 (10)	0.0446 (9)	−0.0008 (8)	−0.0022 (8)	−0.0056 (8)
C9	0.0560 (11)	0.0481 (9)	0.0471 (9)	−0.0079 (9)	0.0045 (8)	−0.0071 (8)
C10	0.0548 (11)	0.0411 (9)	0.0600 (11)	0.0009 (9)	0.0083 (9)	0.0050 (8)
C11	0.0618 (12)	0.0426 (10)	0.0509 (10)	−0.0032 (8)	0.0049 (9)	0.0051 (8)
C12	0.0543 (11)	0.0470 (10)	0.0531 (10)	−0.0119 (9)	0.0061 (8)	−0.0105 (8)
C13	0.0464 (10)	0.0724 (13)	0.0602 (11)	−0.0001 (10)	−0.0044 (9)	−0.0115 (10)
C14	0.0522 (11)	0.0780 (13)	0.0480 (10)	−0.0026 (10)	−0.0083 (8)	−0.0081 (9)
O2	0.0727 (10)	0.0735 (9)	0.0583 (8)	0.0002 (8)	0.0169 (7)	−0.0080 (7)

### Geometric parameters (Å, °)

**Table:**

S1—C8	1.670 (2)	C4—H4A	0.9300
O1—C7	1.223 (2)	C5—C6	1.385 (3)
N1—C7	1.370 (2)	C5—H5A	0.9300
N1—C8	1.388 (2)	C6—C7	1.486 (3)
N1—H1B	0.8600	C9—C14	1.376 (3)
N2—C8	1.328 (2)	C9—C10	1.384 (3)
N2—C9	1.428 (2)	C10—C11	1.376 (3)
N2—H2B	0.8600	C10—H10A	0.9300
C1—C2	1.377 (3)	C11—C12	1.379 (3)
C1—C6	1.385 (3)	C11—H11A	0.9300
C1—H1A	0.9300	C12—O2	1.378 (2)
C2—C3	1.362 (3)	C12—C13	1.381 (3)
C2—H2A	0.9300	C13—C14	1.382 (3)
C3—C4	1.370 (3)	C13—H13A	0.9300
C3—H3A	0.9300	C14—H14A	0.9300
C4—C5	1.377 (3)	O2—H2C	0.8200
			
C7—N1—C8	128.94 (15)	O1—C7—C6	121.81 (17)
C7—N1—H1B	115.5	N1—C7—C6	116.91 (15)
C8—N1—H1B	115.5	N2—C8—N1	115.91 (16)
C8—N2—C9	127.36 (16)	N2—C8—S1	125.54 (15)
C8—N2—H2B	116.3	N1—C8—S1	118.53 (13)
C9—N2—H2B	116.3	C14—C9—C10	119.66 (17)
C2—C1—C6	121.0 (2)	C14—C9—N2	122.90 (17)
C2—C1—H1A	119.5	C10—C9—N2	117.43 (17)
C6—C1—H1A	119.5	C11—C10—C9	120.56 (18)
C3—C2—C1	119.7 (2)	C11—C10—H10A	119.7
C3—C2—H2A	120.2	C9—C10—H10A	119.7
C1—C2—H2A	120.2	C10—C11—C12	119.57 (18)
C2—C3—C4	120.4 (2)	C10—C11—H11A	120.2
C2—C3—H3A	119.8	C12—C11—H11A	120.2
C4—C3—H3A	119.8	O2—C12—C11	122.08 (18)
C3—C4—C5	120.2 (2)	O2—C12—C13	117.68 (18)
C3—C4—H4A	119.9	C11—C12—C13	120.24 (18)
C5—C4—H4A	119.9	C12—C13—C14	119.87 (19)
C4—C5—C6	120.4 (2)	C12—C13—H13A	120.1
C4—C5—H5A	119.8	C14—C13—H13A	120.1
C6—C5—H5A	119.8	C9—C14—C13	120.08 (19)
C1—C6—C5	118.32 (18)	C9—C14—H14A	120.0
C1—C6—C7	123.80 (17)	C13—C14—H14A	120.0
C5—C6—C7	117.87 (17)	C12—O2—H2C	109.5
O1—C7—N1	121.28 (18)		
			
C6—C1—C2—C3	0.2 (3)	C9—N2—C8—S1	7.0 (3)
C1—C2—C3—C4	0.1 (4)	C7—N1—C8—N2	7.5 (3)
C2—C3—C4—C5	−0.5 (4)	C7—N1—C8—S1	−171.15 (16)
C3—C4—C5—C6	0.6 (3)	C8—N2—C9—C14	−49.9 (3)
C2—C1—C6—C5	−0.1 (3)	C8—N2—C9—C10	130.9 (2)
C2—C1—C6—C7	−179.5 (2)	C14—C9—C10—C11	1.6 (3)
C4—C5—C6—C1	−0.3 (3)	N2—C9—C10—C11	−179.13 (16)
C4—C5—C6—C7	179.15 (19)	C9—C10—C11—C12	−1.2 (3)
C8—N1—C7—O1	−7.2 (3)	C10—C11—C12—O2	−179.80 (17)
C8—N1—C7—C6	171.87 (17)	C10—C11—C12—C13	0.4 (3)
C1—C6—C7—O1	−174.8 (2)	O2—C12—C13—C14	−179.95 (18)
C5—C6—C7—O1	5.8 (3)	C11—C12—C13—C14	−0.2 (3)
C1—C6—C7—N1	6.1 (3)	C10—C9—C14—C13	−1.4 (3)
C5—C6—C7—N1	−173.29 (17)	N2—C9—C14—C13	179.44 (19)
C9—N2—C8—N1	−171.58 (17)	C12—C13—C14—C9	0.7 (3)

### Hydrogen-bond geometry (Å, °)

**Table:**

*D*—H···*A*	*D*—H	H···*A*	*D*···*A*	*D*—H···*A*
N2—H2B···O1	0.86	1.95	2.631 (2)	135
N1—H1B···O2^i^	0.86	2.29	3.109 (2)	158
O2—H2C···S1^ii^	0.82	2.53	3.1533 (18)	134
C1—H1A···O2^i^	0.93	2.51	3.429 (3)	172
C11—H11A···O1^iii^	0.93	2.43	3.262 (2)	149

Symmetry codes:  (i) −*x*+5/2, −*y*, *z*+1/2; (ii) −*x*+5/2, −*y*, *z*−1/2; (iii) *x*+1/2, −*y*+1/2, −*z*+1.
